# Adherence to Antiretroviral Treatment and Correlation with Risk of Hospitalization among Commercially Insured HIV Patients in the United States

**DOI:** 10.1371/journal.pone.0031591

**Published:** 2012-02-24

**Authors:** Paul E. Sax, Juliana L. Meyers, Michael Mugavero, Keith L. Davis

**Affiliations:** 1 Brigham and Women's Hospital, Boston, Massachusetts, United States of America; 2 RTI Health Solutions, Research Triangle Park, North Carolina, United States of America; 3 University of Alabama, Birmingham, Alabama, United States of America; Yale University School of Medicine, United States of America

## Abstract

**Purpose:**

A lower daily pill burden may improve adherence to antiretroviral treatment (ART) and clinical outcomes in patients with human immunodeficiency virus (HIV). This study assessed differences in adherence using the number of pills taken per day, and evaluated how adherence correlated with hospitalization.

**Methodology:**

Commercially insured patients in the LifeLink database with an HIV diagnosis (*International Classification of Diseases, 9th Revision, Clinical Modification* code 042.xx) between 6/1/2006 and 12/31/2008 and receipt of a complete ART regimen were selected for inclusion. Patients were grouped according to their daily pill count and remained on ART for at least 60 days. Outcomes included adherence and rates of hospitalization. Adherence was measured as the proportion of days between the start and end of the regimen in which the patient maintained supply of all initiated ART components. Logistic regressions assessed the relationship between pills per day, adherence, and hospitalization, controlling for demographics, comorbidities, and ART-naïve (vs. experienced) status.

**Results:**

7,073 patients met the study inclusion criteria, and 33.4%, 5.8%, and 60.8% received an ART regimen comprising one, two, or three or more pills per day, respectively. Regression analysis showed patients receiving a single pill per day were significantly more likely to reach a 95% adherence threshold versus patients receiving three or more pills per day (odds ratio [OR] = 1.59; *P*<0.001). Regardless of the number of pills received per day, patients were over 40% less likely to have a hospitalization if they were adherent to therapy (OR = 0.57; *P*<0.001). Patients receiving a single pill per day were 24% less likely to have a hospitalization versus patients receiving three or more pills per day (OR = 0.76; *P* = 0.003).

**Conclusions:**

ART consisting of a single pill per day was associated with significantly better adherence and lower risk of hospitalization in patients with HIV compared to patients receiving three or more pills per day.

## Introduction

Adherence to antiretroviral therapy (ART) is important for achieving optimal clinical outcomes in individuals with human immunodeficiency virus (HIV) or acquired immune deficiency syndrome (AIDS). Patients with poor adherence to ART are at an increased risk for incomplete viral suppression, disease progression, and death [1]–[5]. Combination ART initially was characterized by high pill burdens and multiple daily doses. More recently, fixed-dose combinations and agents with prolonged half-lives have allowed for markedly simpler regimens: notably, all the preferred initial treatments for HIV or AIDS under current treatment guidelines consist of four or fewer pills per day with once- or twice-daily dosing [6], [7].

Clinical trials and cohort studies suggest that an ART regimen composed of a single pill per day may significantly improve adherence, patient satisfaction, and virological outcomes [8]–[11]. In prospective studies of patients switching to single-pill treatments, adherence to an ART regimen has been estimated to be up to 98% in prospective trials [8]–[10]. While adherence was high in these patients prior to the switch, it numerically improved in some of the studies after switching to fewer daily pills. Furthermore, among homeless or marginally housed patients, those receiving an ART regimen composed of a single pill per day had better virologic outcomes and a 26% increase in adherence, compared with patients receiving other multi-pill per day regimens [11]. Although these studies support the adherence benefit of an ART regimen composed of a single pill per day, limited data exist on individuals with HIV or AIDS receiving this regimen in clinical practice. Therefore, the primary objective of this retrospective database analysis was to assess the effect of ART as a single pill per day on adherence and hospitalization in a large population of managed care enrollees in the United States who received treatment for HIV or AIDS.

## Methods

Data for this analysis were taken from the LifeLink database (formerly the PharMetrics Integrated Outcomes database), a national insurance claims database encompassing 95 United States managed care organizations covering over 61 million lives between 1997 and 2008. This database contains patient-level demographics, periods of health plan enrollment, primary and secondary diagnoses, and detailed information about hospitalizations and therapeutic procedures, inpatient and outpatient physician services, and prescription drug use. In compliance with the Health Insurance and Portability and Accountability Act of 1996, all data were de-identified to protect the privacy of individual patients, physicians, and hospitals. Because the data were retrospective, preexisting, and de-identified, RTI International's institutional review board determined that this study met all criteria for exemption.

Patients were selected for inclusion if they received at least one HIV or AIDS diagnosis (*International Classification of Diseases, 9th Revision, Clinical Modification* [ICD-9-CM] code 042.xx) between June 1, 2006, and December 31, 2008. Patients also were required to have evidence of receipt of a complete ART regimen, defined as two nucleoside/nucleotide reverse transcriptase inhibitors plus a third agent (i.e., another nucleoside/nucleotide reverse transcriptase inhibitor, a nonnucleoside/nucleotide reverse transcriptase inhibitor, a protease inhibitor [PI], a chemokine receipt 5 antagonist, or an integrase inhibitor). ART agents were identified in the claims database by using National Drug Codes associated with relevant generic and brand names. Further, patients were required to remain on the complete regimen for at least 60 days following first observed uptake and to have evidence of continuous enrollment in their health plan during this period.

Patients were grouped into three mutually exclusive cohorts according to the daily pill count of their complete ART regimen. Patients were assigned to the single-pill-per-day cohort if they received an ART regimen consisting of a single pill at any point during the selection window, regardless of prior or subsequent use of other regimens. At the time of this study, only coformulated tenofovir/emtricitabine/efavirenz was available as a single pill per day. Patients were assigned to the two-pills-per-day cohort if they received a regimen consisting of two pills per day at any point during the selection window and if they did not receive a regimen consisting of a single pill per day at any point during the selection window. Finally, patients were assigned to the three-or-more-pills-per-day cohort if they received a regimen consisting of three or more pills per day at any point during the selection window and if they did not receive a regimen consisting of either a single pill per day or two pills per day at any point during the selection window.

Patients were followed from the start of their complete ART regimen (which defined the study index date) until the earliest date of regimen discontinuation, disenrollment from the health plan, or the end of the database (i.e., March 31, 2009). Discontinuation was defined as 90 consecutive days in which no refills were observed for any component of the regimen.

Patient characteristics measured at the index date included age, sex, geographic region, health insurance coverage, and ART classes received (i.e., nucleoside/nucleotide reverse transcriptase inhibitors, nonnucleoside/nucleotide reverse transcriptase inhibitors, PIs, ritonavir boosting, and other therapies). The presence of comorbid medical conditions other than HIV or AIDS was assessed during the 6-month pre-index period using an established algorithm, the Charlson Comorbidity Index (CCI) score [12]. This score is made up of 17 comorbidities (defined by ICD-9-CM diagnosis and procedure codes), such as myocardial infarction and chronic pulmonary disease, which are weighted to correspond to the severity of the comorbid condition of interest. A higher comorbidity score represents a higher overall comorbidity burden during the pre-index period. Additionally, the incidence of other concomitant mental disorders (ICD-9-CM codes 306.xx to 319.xx) and drug and alcohol abuse (ICD-9-CM codes 292.xx and 303.xx to 305.xx) during the 6-month pre-index period also were assessed.

Medication adherence was assessed using the medication possession ratio (MPR), which has been shown to be the most widely adopted measure in published claims-based analyses (57% of all studies) of medication adherence [13] and has been used in studies of ART adherence among individuals with HIV [14]. For each patient, the MPR was calculated over the period in which the patient remained exposed to his or her ART regimen. The MPR, which is a proxy for refill compliance, generally measures the proportion of the ART exposure period in which supply was maintained for all ART components comprising the regimen. Specifically, MPR was calculated as the number of filled prescription days for all ART regimen components (using the days supplied in the pharmacy claims) divided by the number of days from the first observed prescription in the regimen through the earliest of either the exhaustion of the days supplied of the last observed prescription or the end of follow-up. For patients in either of the two- or three-or-more-pill-per-day cohorts, late refills and resulting days of missing supply for only one ART component were factored against their adherence measurement. Patients in the three-or-more pills-per-day cohort with a supply for only two of their ART components on a given day, for example, were considered to have zero adherence for that day. In addition to reporting the mean (standard deviation) MPR achieved, we also reported the numbers and percentages of patients achieving various adherence thresholds (i.e., MPRs of 0.95, 0.90, 0.85, and 0.80, corresponding to 95%, 90%, 85%, and 80% adherence, respectively).

Hospitalizations were identified from the claims database using relevant place of service codes. Hospitalizations were observed from the index date until the earliest date of regimen discontinuation, end of enrollment in the health plan, or end of the database. The number and percentage of patients with at least one hospitalization were reported, along with the mean (standard deviation) number of hospitalizations.

All analyses were carried out using SAS (Version 9; Cary, North Carolina) statistical software. Descriptive analyses were conducted for all outcome measures and included means and standard deviations for continuous variables of interest (e.g., MPR) and frequency distributions of categorical variables of interest (e.g., geographic region). All descriptive analyses were stratified by each pill-count cohort.

Multivariate logistic regression analyses were conducted to assess the relationship between the number of pills per day, adherence, and hospitalization. The dependent variables included binary indicators for achieving an MPR threshold of 0.95 (i.e., 95% adherence) and whether the patient was hospitalized during exposure to the ART regimen. Independent variables included in each logistic model were as follows: treatment regimen received (i.e., single pill per day and two pills per day vs. the reference category of three or more pills per day), age, sex, geographic region, health plan type, payer type, CCI score, treatment-naïve status, pre-index presence of mental health disorders, and pre-index presence of alcohol or drug abuse disorders. Since the number of pills received per day and achieving a 95% adherence threshold were likely proxies for each other, two separate models assessing hospitalization risk were estimated. Odds ratios (ORs) were reported for all covariates.

## Results

**Figure 1:**
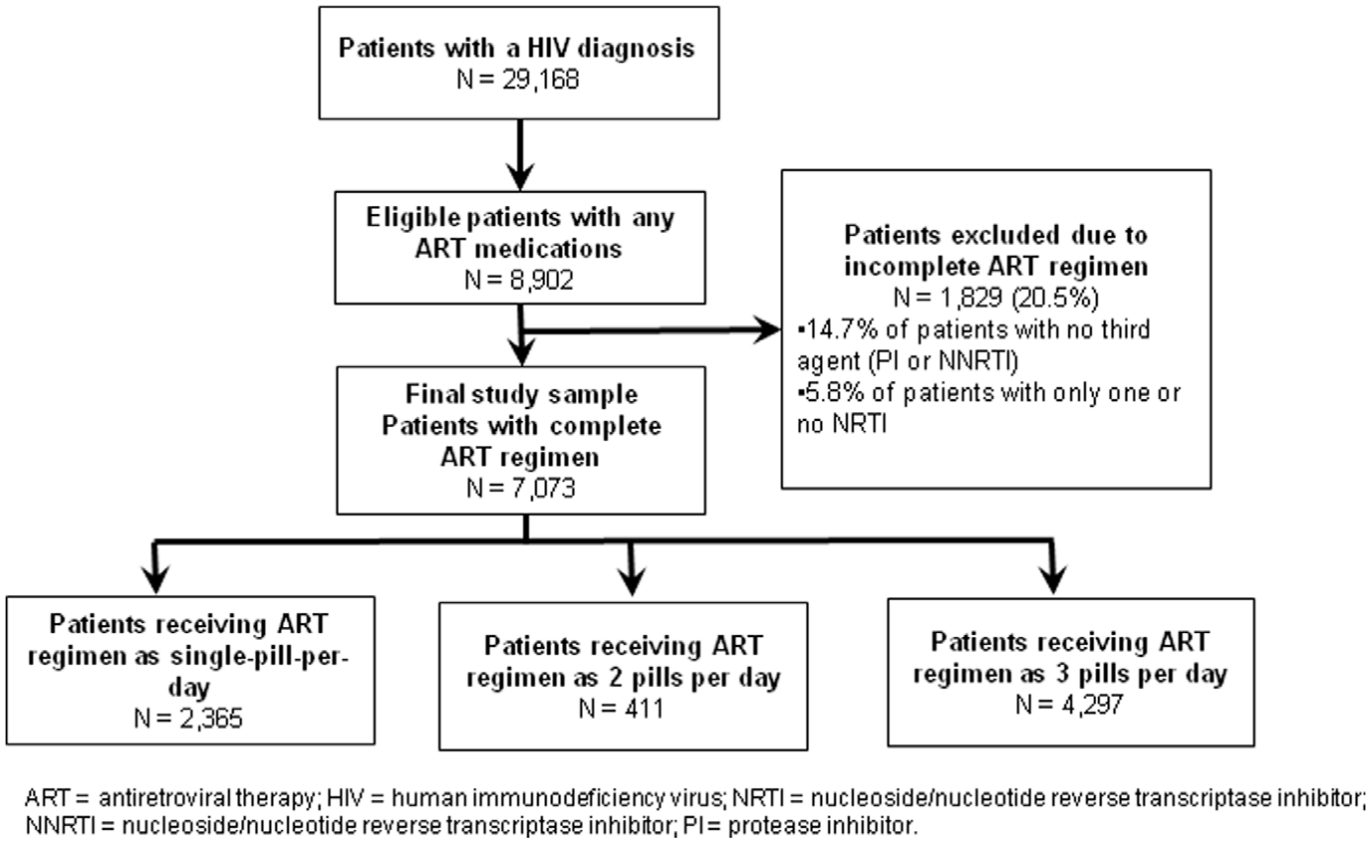
Sample Selection Flow Chart.

A total of 7,073 patients met the selection criteria (Figure 1). Most patients received their ART regimen as three or more pills per day (60.8% of patients) or as a single pill per day (33.4% of patients). On average, patients were approximately 45 years of age. Approximately one-fifth of patients were female (Table 1). Across all three cohorts, most patients were covered by commercial health insurance, and the average CCI score was approximately the same. Furthermore, the incidence of concomitant mental disorders and drug and alcohol abuse diagnoses did not vary substantially by cohort. Patients tended to remain on the observed ART regimen for approximately 13 months, and patients receiving three or more pills per day had a mean regimen duration that was approximately 1.5 months longer than that observed for patients receiving fewer than three pills per day (i.e., mean regimen duration of 433 days among patients receiving three or more pills per day vs. 379 and 386 days among patients receiving a single pill per day and two pills per day, respectively). Forty-two percent of patients receiving a single pill per day were treatment naïve, compared with 25% of patients receiving two pills per day and 20% of patients receiving three or more pills per day.

**Table 1:** Characteristics of the Study Sample, by Cohort.

	Patient Cohort					
	Single Pill Per Day		Two Pills Per Day		Three or More Pills Per Day	
Total sample (N)	2,365		411		4,297	
Female (N, %)	481	20.34%	77	18.73%	990	23.04%
Age (Years) (N, %)						
<34	325	13.74%	48	11.68%	393	9.15%
35–44	804	34.00%	145	35.28%	1,394	32.44%
45–54	862	36.45%	148	36.01%	1,782	41.47%
≥55	374	36.60%	70	17.03%	728	16.94%
Mean (SD)	44.66	(9.35)	44.96	(9.01)	45.89	(9.02)
Geographic region (N, %)						
East	855	36.15%	138	33.58%	1,352	31.46%
South	697	29.47%	120	29.20%	1,541	35.86%
Midwest	566	23.93%	94	22.87%	907	21.11%
West	247	10.44%	59	14.36%	497	11.57%
Health plan type (N, %)						
HMO	742	31.37%	111	27.01%	1,221	28.42%
PPO	1,138	48.12%	209	50.85%	2,044	47.57%
Other/missing	485	20.50%	91	22.14%	1,032	24.02%
Payer type (N, %)						
Commercial	2,033	85.96%	357	86.86%	3,696	86.01%
Self	220	9.30%	36	8.76%	405	9.43%
Other/missing	112	4.73%	18	4.38%	196	4.56%
Mean (SD) Charlson Comorbidity score^a^	0.51	(1.30)	0.63	(1.37)	0.42	(1.15)
Concomitant mental disorders (N, %)	168	7.10%	25	6.10%	219	5.10%
Concomitant drug/alcohol abuse (N, %)	345	14.60%	56	13.60%	619	14.40%
Treatment-naïve patients (N, %)	988	41.78%	101	24.57%	851	19.80%
ART classes received at index						
Nucleoside/nucleotide reverse transcriptase inhibitors	2,365	100.00%	411	100.00%	4,297	100.00%
Pharmacokinetic enhancers	0	0.00%	0	0.00%	1,240	28.86%
Nonnucleoside reverse transcriptase inhibitors	2,365	100.00%	411	100.00%	1,411	32.84%
Protease inhibitors	0	0.00%	0	0.00%	2,808	65.35%
Entry inhibitors	0	0.00%	0	0.00%	58	1.35%
Integrase inhibitors	0	0.00%	0	0.00%	17	0.40%
Mean (SD) regimen duration (days)	378.58	(253.69)	385.70	(290.49)	433.22	(299.90)

ART = antiretroviral therapy; HMO = health maintenance organization; POS = point of service; PPO = preferred provider organization; SD = standard deviation.

a Evaluated in the 6-month pre-index period.

Patients receiving a single pill per day had significantly better adherence when compared with patients receiving multiple pills per day. Approximately 47% of patients receiving a single pill per day achieved 95% adherence or greater, compared with 41% of patients receiving two pills per day and 34% of patients receiving three or more pills per day (*P* = 0.019 for single pill vs. two pills; *P*<0.001 for single pill vs. three or more pills). Mean (standard deviation) MPR was 0.92 (0.09) among patients receiving a single pill per day, 0.90 (0.10) among patients receiving two pills per day, and 0.90 (0.09) among patients receiving three or more pills per day (*P*<0.01 for single pill vs. two pills and for single pill vs. three or more pills) (Table 2).

**Table 2:** Adherence to ART Regimens, by Cohort.

	Patient Cohort						*P* Values^a^		
	Single Pill Per Day		Two Pills Per Day		Three or More Pills Per Day		Single Pill Per Day vs. Two Pills Per Day	Single Pill Per Day vs. Three or More Pills Per Day	Two Pills Per Day vs. Three or More Pills Per Day
MPR									
Mean (SD)	0.92	(0.09)	0.90	(0.10)	0.90	(0.09)	0.006	<0.0001	0.188
Median	0.95		0.94		0.92		0.0020	<0.0001	0.004
Minimum, maximum	0.50	1.00	0.17	1.00	0.20	1.00			
0.95 threshold (N, %)									
MPR≥0.95	1,114	47.10%	168	40.88%	1,468	34.16%	0.0194	<0.0001	0.006
MPR<0.95	1,251	52.90%	243	59.12%	2,829	65.84%			
0.90 threshold (N, %)									
MPR≥0.90	1,698	71.80%	281	68.37%	2,664	62.00%	0.1563	<0.0001	0.011
MPR<0.90	667	28.20%	130	31.63%	1,633	38.00%			
0.85 threshold (N, %)									
MPR≥0.85	1,963	83.00%	319	77.62%	3,316	77.17%	0.0084	<0.0001	0.837
MPR<0.85	402	17.00%	92	22.38%	981	22.83%			
0.80 threshold (N, %)									
MPR≥0.80	2,128	89.98%	355	86.37%	3,703	86.18%	0.0282	<0.0001	0.911
MPR<0.80	237	10.02%	56	13.63%	594	13.82%			
MPR quintiles (N, %)									
0.8–1	2,128	89.98%	355	86.37%	3,703	86.18%	0.0282	<0.0001	0.911
0.6–0.79	210	8.88%	51	12.41%	536	12.47%	0.0236	<0.0001	0.970
0.4–0.59	27	1.14%	3	0.73%	56	1.30%	0.4562	0.5694	0.318
0.2–0.39	0	0.00%	1	0.24%	1	0.02%	—	—	—
<0.2	0	0.00%	1	0.24%	1	0.02%	—	—	—

ART = antiretroviral therapy; MPR = medication possession ratio; SD = standard deviation.

a *P* values calculated from Students t-test for mean MPR, Wilcoxon rank sum tests for median MPR, and chi-square for distribution by MPR threshold.

Multivariate logistic regression models showed that receiving a single pill per day was associated with a 59% greater likelihood of achieving a 95% adherence threshold, compared with receiving three or more pills per day (OR = 1.587, *P*<0.001). The following were also associated with a greater likelihood of achieving a 95% adherence threshold (Table 3): receiving two pills per day versus three or more pills per day (OR = 1.346, *P* = 0.006), being treatment naïve versus treatment experienced (OR = 1.378, *P*<0.001), and receiving treatment in 2008 versus 2006 (OR = 1.418, *P*≤0.001). The presence of a concomitant drug or alcohol abuse diagnosis during the 6-month pre-index period was found to be associated with a significantly lower likelihood of achieving a 95% adherence threshold (OR = 0.599, *P*<0.001), as was being female (OR = 0.869, *P* = 0.027).

**Table 3:** Predictors of Achieving a 95% Adherence Threshold to ART, Using Multivariate Logistic Regression.

	Odds Ratio	Lower 95% CI	Upper 95% CI	*P* Value
Treatment regimen (vs. three or more pills per day)				
Single pill per day	1.587	1.415	1.780	<0.001
Two pills per day	1.346	1.091	1.660	0.006
Female (vs. male)	0.869	0.767	0.984	0.027
Age (years) (vs. <35)				
35–44	1.074	0.901	1.279	0.426
45–54	1.136	0.955	1.351	0.151
55–64	1.250	1.025	1.523	0.027
≥65	1.671	0.470	5.944	0.428
Geographic region (vs. East)				
South	0.956	0.830	1.101	0.530
Midwest	0.909	0.786	1.050	0.194
West	0.619	0.509	0.752	<0.001
Health plan type (vs. HMO)				
PPO	1.095	0.962	1.246	0.169
POS	0.879	0.735	1.051	0.156
Indemnity	1.057	0.829	1.348	0.653
Consumer directed	1.257	0.989	1.597	0.061
Other/missing	1.062	0.613	1.841	0.829
Payer type (vs. commercial)				
Medicaid	0.529	0.375	0.745	<0.001
Medicare	0.885	0.575	1.361	0.577
Self	0.691	0.564	0.848	<0.001
Other/missing	2.046	0.609	6.873	0.247
Charlson Comorbidity score (vs. ≤1)				
Between 1 and 2	1.022	0.785	1.330	0.873
Between 2 and 3	0.949	0.728	1.238	0.701
>3	0.863	0.647	1.152	0.319
Treatment naïve pre-index (vs. treatment experienced)	1.378	1.209	1.57	<0.001
Had a mental disorder diagnosis	1.033	0.895	1.192	0.655
Had a drug or alcohol abuse diagnosis	0.599	0.476	0.753	<0.001
Received treatment in 2007 (vs. 2006)	0.926	0.798	1.076	0.316
Received treatment in 2008 (vs. 2006)	1.418	1.194	1.685	<0.001

ART = antiretroviral therapy; CI = confidence interval; HMO = health maintenance organization; POS = point of service; PPO = preferred provider organization.

**Figure 2:**
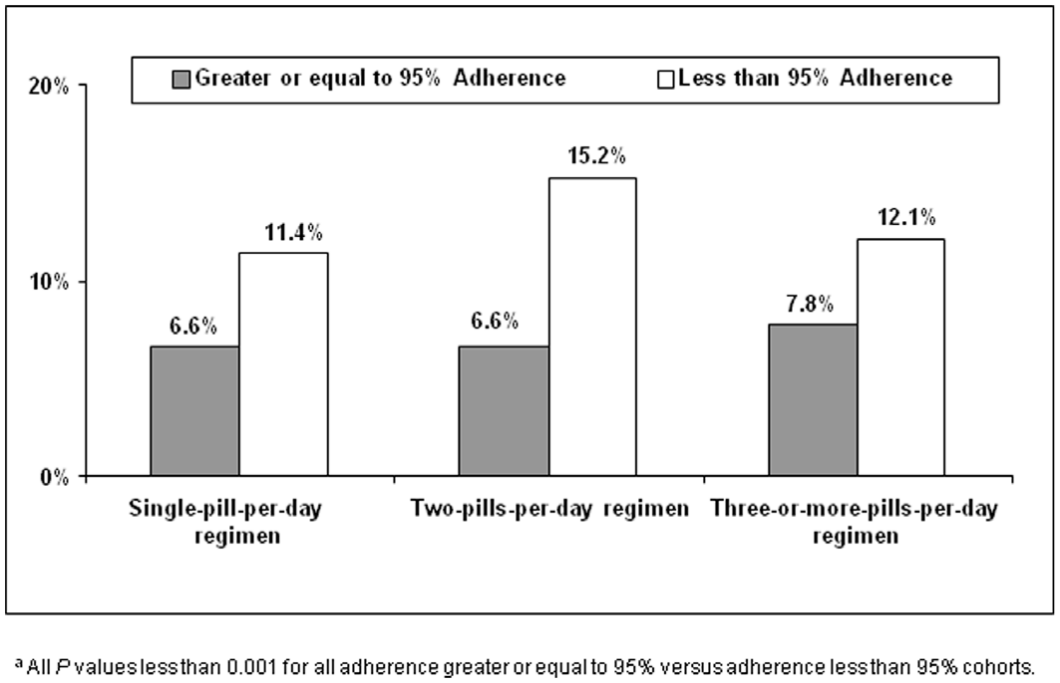
Frequency of Hospitalizations, by Cohort^a^.

Patients who achieved a 95% adherence threshold had a significantly (*P*<0.001) lower rate of hospitalization, regardless of pill burden, compared with patients who did not achieve a 95% adherence threshold (Figure 2). Specifically, among patients who received a single pill per day, 6.6% of patients who achieved a 95% adherence threshold had at least one hospitalization, compared with 11.4% of patients who did not achieve a 95% adherence threshold. Similarly, among patients who received two pills per day, 6.6% of patients who achieved a 95% adherence threshold had at least one hospitalization, compared with 15.2% of patients who did not achieve a 95% adherence threshold. Among patients who received three or more pills per day, 7.8% of patients who achieved a 95% adherence threshold had at least one hospitalization, compared with 12.1% of patients who did not achieve a 95% adherence threshold.

Our multivariate logistic regression models showed that achieving a 95% adherence threshold was associated with a significantly lower risk of hospitalization when compared with not achieving a 95% adherence threshold (OR = 0.572, *P*<0.001) (Table 4). When we controlled for adherence, we observed that patients were significantly more likely to be hospitalized if they had a concomitant mental disorder diagnosis versus no concomitant mental disorder diagnosis (OR = 1.388, *P* = 0.002), had a concomitant drug or alcohol abuse diagnosis versus no concomitant drug or alcohol abuse diagnosis (OR = 1.884, *P*<0.001), were female versus male (OR = 1.249, *P* = 0.022), were covered by Medicare versus commercial insurance (OR = 2.156, *P* = 0.005), were self-insured versus covered by commercial insurance (OR = 1.412, *P* = 0.025), or had a CCI score greater than 1 (OR increased with increasing CCI score, from 2.265 among patients with a CCI between 1 and 2 to 5.094 among patients with a CCI greater than 3, all *P*<0.001).

**Table 4:** Predictors of Hospitalization, Using Multivariate Logistic Regression and Controlling for Adherence Threshold.

	Odds Ratio	Lower 95% CI	Upper 95% CI	*P* Value
Achieved a 95% adherence threshold	0.572	0.479	0.682	<0.001
Female (vs. male)	1.249	1.032	1.510	0.022
Age (years) (vs. <35)				
35–44	0.815	0.619	1.072	0.143
45–54	0.829	0.632	1.087	0.175
55–64	1.013	0.746	1.375	0.934
≥65	0.558	0.065	4.746	0.593
Geographic region (vs. East)				
South	1.101	0.873	1.390	0.416
Midwest	1.130	0.893	1.429	0.308
West	0.964	0.704	1.320	0.817
Health plan type (vs. HMO)				
PPO	1.199	0.964	1.491	0.103
POS	1.271	0.956	1.692	0.099
Indemnity	1.285	0.871	1.897	0.206
Consumer directed	0.612	0.364	1.029	0.064
Other/missing	0.871	0.374	2.026	0.748
Payer type (vs. commercial)				
Medicaid	1.080	0.674	1.730	0.748
Medicare	2.156	1.260	3.692	0.005
Self	1.412	1.044	1.910	0.025
Other/missing	2.336	0.460	11.862	0.306
Charlson Comorbidity score (vs. ≤1)				
Between 1 and 2	2.265	1.609	3.188	<0.001
Between 2 and 3	2.446	1.762	3.396	<0.001
Greater than 3	5.094	3.759	6.904	<0.001
Treatment naïve pre-index (vs. treatment experienced)	1.160	0.972	1.385	0.100
Had a mental disorder diagnosis	1.388	1.130	1.705	0.002
Had a drug or alcohol abuse diagnosis	1.884	1.433	2.477	<0.001

CI = confidence interval; HMO = health maintenance organization; POS = point of service; PPO = preferred provider organization.

Similarly, in separate logistic models, patients who received a single pill per day had a significantly lower likelihood of being hospitalized, compared with patients receiving three or more pills per day (OR = 0.764, *P* = 0.003) (Table 5). When controlling for treatment regimen received, we observed that patients were significantly more likely to be hospitalized if they had a concomitant drug or alcohol abuse diagnosis versus having no concomitant drug or alcohol abuse diagnosis (OR = 2.023, *P*<0.001), had a concomitant mental disorder diagnosis versus having no concomitant mental disorder diagnosis (OR = 1.372, *P* = 0.003), were female versus male (OR = 1.260, *P* = 0.017), were covered by Medicare versus commercial insurance (OR = 2.173, *P* = 0.004), were self-insured versus covered by commercial insurance (OR = 1.463, *P* = 0.013), or had a CCI score greater than 1 (OR increased with increasing CCI score, from 2.279 among patients with a CCI score between 1 and 2 to 5.147 among patients with a CCI score greater than 3, all *P*<0.001).

**Table 5:** Predictors of Hospitalization, Using Multivariate Logistic Regression and Controlling for Treatment Regimen Received.

	Odds Ratio	Lower 95% CI	Upper 95% CI	*P* Value
Treatment regimen (vs. three or more pills per day)				
Single pill per day	0.764	0.638	0.915	0.003
Two pills per day	1.001	0.721	1.388	0.997
Female (vs. male)	1.260	1.042	1.524	0.017
Age (years) (vs. <35)				
35–44	0.801	0.609	1.054	0.113
45–54	0.799	0.609	1.049	0.106
55–64	0.972	0.717	1.319	0.856
≥65	0.527	0.062	4.467	0.557
Geographic region (vs. East)				
South	1.100	0.871	1.387	0.424
Midwest	1.142	0.903	1.443	0.267
West	1.006	0.735	1.376	0.972
Health plan type (vs. HMO)				
PPO	1.176	0.947	1.462	0.143
POS	1.274	0.958	1.694	0.096
Indemnity	1.233	0.835	1.819	0.292
Consumer directed	0.590	0.351	0.992	0.046
Other/missing	0.871	0.378	2.007	0.745
Payer type (vs. commercial)				
Medicaid	1.134	0.709	1.813	0.599
Medicare	2.173	1.275	3.706	0.004
Self	1.463	1.084	1.976	0.013
Other/missing	2.106	0.418	10.618	0.367
Charlson Comorbidity score (vs. ≤1)				
Between 1 and 2	2.279	1.621	3.205	<0.001
Between 2 and 3	2.490	1.796	3.452	<0.001
Greater than 3	5.147	3.805	6.963	<0.001
Treatment naïve pre-index (vs. treatment experienced)	1.153	0.963	1.380	0.121
Had a mental disorder diagnosis	1.372	1.118	1.684	0.003
Had a drug or alcohol abuse diagnosis	2.023	1.541	2.654	<0.001

CI = confidence interval; HMO = health maintenance organization; POS = point of service; PPO = preferred provider organization.

**Figure 3:**
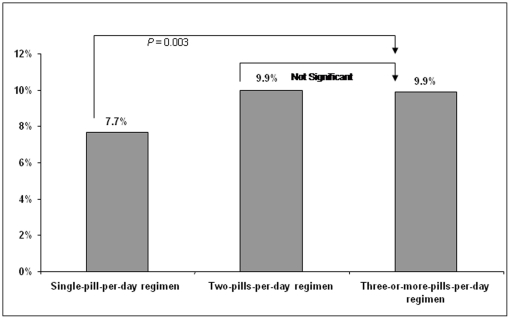
Adjusted Rate of Hospitalization, by Cohort.

In the estimated logistic regression analysis that controlled for the number of pills received per day and clinical and demographic characteristics, the adjusted rate of hospitalization was found to be significantly lower for patients receiving a single pill per day compared with patients receiving three or more pills per day (i.e., 7.7% of patients receiving a single pill per day were predicted to be hospitalized vs. 9.9% of patients receiving three or more pills per day) (Figure 3). Further, there was a 24% lower risk of hospitalization among patients receiving a single pill per day compared with patients receiving three or more pills per day.

## Discussion

This retrospective database analysis examined adherence to ART regimens among patients with HIV or AIDS. The rate of hospitalization and the predictors of hospitalization also were assessed. This study found that patients receiving a single pill per day had significantly better adherence and consistently achieved higher adherence thresholds than patients receiving multiple pills per day. Additionally, patients who achieved a 95% adherence threshold had a significantly lower rate of hospitalization (*P*<0.001) compared with patients who were nonadherent to therapy, regardless of their pill burden. Also, patients who received a single pill per day were significantly less likely to be hospitalized than patients who received three or more pills per day.

The guidelines of the United States Department of Health and Human Services and the International AIDS Society-USA currently list four regimens as preferred for initial therapy. All four contain coformulated tenofovir-emtricitabine, with the remainder of the regimen consisting of 1) efavirenz or 2) ritonavir-boosted atazanavir or 3) ritonavir-boosted darunavir or 4) raltegravir [7]. The first three of these regimens are administered once daily, while the raltegravir-based treatment requires twice-daily dosing. Tenofovir-emtricitabine-efavirenz is the only preferred regimen currently available as a single pill per day; this was the most commonly chosen regimen in our study sample, used in 33% of patients. The atazanavir- and raltegravir-based treatments contain three pills, and darunavir-based treatments contain four. Coformulated abacavir-lamivudine plus efavirenz was the only available two-pill regimen at the time of our study; this is listed as an alternative initial treatment in the above-mentioned guidelines.

Consistent with previous literature, we found that patients in our study generally were adherent to ART, regardless of the number of pills received per day [9], [10]. Patients who received treatment as a single pill per day had significantly better adherence than patients who received multiple pills per day. Furthermore, this difference (approximately 2.2%) was in line with the increased adherence effect reported by Parienti and colleagues in a meta-analysis of 11 studies examining the difference in adherence between once-daily dosed and twice-daily dosed regimens [15]. Specifically, Parienti and colleagues reported a 2.9% increase in adherence with a once-daily dosed regimen compared with a twice-daily dosed regimen [15].

Among patients receiving complex, multi-pill regimens, adherence estimates typically have ranged from 60% to 70% [5], [16], [17]. Achieving optimal outcomes in HIV/AIDS treatment, however, requires a much higher, sustained level of adherence throughout a patient's lifetime [18]. Studies conducted on patients receiving older ART regimens have suggested that an ART adherence rate of at least 95% was required to achieve a lower risk of virologic failure, fewer hospital days, and reduced morbidity and mortality in patients with HIV or AIDS [5], [16], [19]. Simpler regimens with longer half-lives may allow for a lesser degree of adherence [20], but the goal of maximum adherence remains an important one in clinical care. Therefore, reducing pill burden to a single pill per day may improve adherence to ART and reduce the risk of hospitalization for patients with HIV or AIDS [15]. On the basis of these and other benefits (patient preference, fewer copays), other coformulated combinations are under investigation [21], [22].

Our adherence estimates for patients receiving a single pill per day were slightly lower than previously reported estimates (i.e., 0.92 reported in the present analysis vs. 0.96 reported by both Hodder et al. and Airoldi et al.) [9], [10]. The differences in adherence estimates may be due to the fact that both the Hodder and Airoldi studies examined patients in a clinical trial setting, while our patients were identified using health care claims (i.e., a real-world treatment setting). Additionally, as expected, our estimate for patients receiving a single pill per day were higher than the estimate (0.86) previously reported by Bangsberg and colleagues in a homeless and marginally housed population [23].

Similar to previous studies [24], [25], we found that patients who were adherent to therapy were less likely to have a hospital stay. Additionally, this study also attempted to control for various baseline differences in the study populations and the effects these differences may have had on rates of adherence and hospitalization. Specifically, multivariate logistic regressions were undertaken that controlled for patient demographics, treatment characteristics (i.e., treatment naïve vs. experienced, type of ART received, year ART received), and clinical characteristics (i.e., CCI score, concomitant mental disorder, drug and alcohol abuse diagnoses). We found a number of factors were associated with an increased risk of poor adherence, including having Medicaid, Medicare, or no insurance; having a CCI score greater than 3; having a drug or alcohol abuse diagnosis; and being treatment experienced. Similarly, having Medicare or no insurance, a CCI score greater than 1, or a concomitant mental disorder or drug or alcohol abuse diagnosis was associated with an increased risk of hospitalization.

In the logistic regression model assessing predictors of achieving a 95% adherence level as a function of the number of pills received per day, the comparator cohort was patients receiving three or more pills per day. Separate analyses were conducted using the single-pill-per-day cohort as the comparator. When the single-pill-per-day variable was used as the comparator, no significant differences based on the multivariate model were observed between patients receiving a single pill per day and patients receiving two pills per day. However, significant differences were observed for the unadjusted mean and median adherence values, along with the percentage of patients achieving a 95% adherence level, among patients receiving a single pill per day versus patients receiving two pills per day. We hypothesize that the lack of significance based on the multivariate models was likely due to the small number of patients who received two pills per day.

Our study has several limitations common to observational claims database analyses. Adherence was measured from filled prescriptions (as opposed to the number of tablets ingested); however, studies have suggested that pharmacy refill rates are a good proxy for actual medication adherence [26].

Because patients were not randomized to the different treatments, we cannot exclude unmeasured confounding factors that may have influenced our outcomes. Among the most important of these factors in this study is that multiple trials have shown resistance or virologic failure is significantly less common in boosted PI treatments than in nonnucleoside/nucleotide reverse transcriptase inhibitor-based treatments [27], [28]. As such, clinicians may have preferentially prescribed the boosted-PI–containing regimens (all of which contain three or more pills per day) to their less-adherent patients. Although we attempted to control for some of these variables through use of multivariable models that include some of these factors (substance abuse and psychiatric diagnoses), residual confounding may remain.

A large proportion of HIV-treated individuals (20.5% of the total HIV-treated population) were excluded from the analysis due to receiving incomplete ART regimens; we did not have sufficient data on these patients to explain why their regimens were incomplete. However, a previous study found that physician medication errors are somewhat common in individuals with HIV, with the most common error occurring for boosted PIs (estimated at 5.3% of patients), which may explain some of the incomplete regimens observed in our analysis [29]. Increased adoption of fixed-dose combinations as part of HIV treatment may help to alleviate the issue of incomplete regimens.

This study examined only three years of data, and therefore may not account for secular trends in adherence counseling and the decision for starting treatment. Since this study used retrospective claims data, it was not possible to determine the daily dosing frequency over which those pills were distributed (e.g., twice daily, three times daily). During the study period, the only available single-pill ART regimen was coformulated efavirenz/emtricitabine/tenofovir disoproxil fumarate, and abacavir/lamivudine plus efavirenz was the only two-pill-per-day treatment. It is possible that these results would not be generalizable to other one- and two-pill-per-day treatments if they had different efficacy and toxicity profiles. Finally, because managed care data were used, results from this study may not be applicable to Medicaid or uninsured populations.

In summary, this study found that patients who receive ART as a single pill per day are significantly more likely to be highly adherent to therapy. Furthermore, receiving a single pill per day was associated with a lower risk of hospitalization when compared with receiving multiple pills per day. Although this study could not assess causality, it did show that receiving ART as a single pill per day was associated with potential clinical and economic benefits.
